# Combining Organic Fertilizer With Controlled-Release Urea to Reduce Nitrogen Leaching and Promote Wheat Yields

**DOI:** 10.3389/fpls.2021.802137

**Published:** 2021-12-24

**Authors:** Xiuyi Yang, Chao Zhang, Xiaoli Ma, Qianjin Liu, Juan An, Shujian Xu, Xingyuan Xie, Jibiao Geng

**Affiliations:** ^1^Shandong Provincial Key Laboratory of Water and Soil Conservation and Environmental Protection, College of Resources and Environment/College of Agricultural and Forestry Science, Linyi University, Linyi, China; ^2^Jinyimeng Group Co. Ltd., Linshu, China; ^3^Linyi Inspection and Testing Center, Linyi, China

**Keywords:** controlled-release urea, organic fertilizer, nitrogen leaching, nitrogen use efficiency, wheat yield

## Abstract

Soil deterioration, low nitrogen use efficiency (NUE), and environmental risks caused by excessive chemical N fertilizer use are key factors restricting sustainable agriculture. It is extremely critical to develop effective N management strategies that consider both environmental and agronomic benefits. From 2017 to 2019, a field experiment was conducted to assess the effects of combinations of organic fertilizers (OF, provided at 30, 50, and 70% of the total applied N) and controlled-release urea (CU) on the NUE, N leaching and wheat yield compared with the effects of urea and CU. The results suggested that OF released N slowly in the early stage and showed a significant residual effect, while CU released N quickly in the first 2 months. The OF substitutes with 30–50% CU increased wheat yield by 4.2–9.2%, while the 70%OF+30%CU treatment showed no significant difference relative to the urea treatment. The average maximum apparent NUE recovery (50.4%) was achieved under the 50%OF+50%CU treatment, but the partial factor productivity was not affected by the N type. As the OF application rate increased, the total carbon content increased, and the total N value decreased. The ${\mathrm{NO}}_{3}^{-}$ -N and ${\mathrm{NH}}_{4}^{+}$ -N concentrations in the OF+CU treatments were lower before the jointing stage but higher from the grain-filling to mature stages than those in the urea treatment. ${\mathrm{NO}}_{3}^{-}$ -N and ${\mathrm{NH}}_{4}^{+}$ -N were mainly concentrated in the 0–60-cm layer soil by OF substitution, and N leaching to the 60–100-cm soil layer was significantly reduced. Hence, the results suggest that the combination of 30–50% OF with CU synchronizes absorption with availability due to a period of increased N availability in soils and proved to be the best strategy for simultaneously increasing wheat production and reducing N leaching.

## Introduction

Wheat (*Triticum aestivum* L.) is the third most cultivated cereal in the world, and optimizing fertilization practices is the main factor controlling the maintenance of wheat yields and protecting the environment (Hao et al., 2020). China is the largest producer and consumer of agricultural chemicals, amounting to 30% of global fertilizer use (Akhtar et al., 2020). This booming chemical fertilizer consumption has made it possible to produce sufficient amounts of food to feed the increasing population of China (Liu et al., 2013). However, the overuse of chemical fertilizers (especially N) and improper fertilization methods have led to diminishing returns of production and other adverse effects (Humber et al., 2016). Earlier N fertilizer application increases the risk of N loss from the root zone by leaching and denitrification. Split- or late-season applications of N fertilizer are common approaches to improve wheat yield and NUE. Biomass was greater under split N application (applied at the tillering and flag leaf stages) than under full N application at tillering (Velasco et al., 2012). Although split fertilizer application requires additional labor, this fertilization method is typically used on crops under traditional agriculture practices (Hickman et al., 2014). However, this fertilization method is currently inappropriate in China, as the worker population is aging and labor shortages for crop-production activities are becoming increasingly severe (Zhou et al., 2017). Thus, balancing the benefits derived from fertilizers with the associated environmental issues is ultimately indispensable.

When urea is used as basal fertilization, the available N is rapidly mineralized for wheat, but less than half of the total N is effectively utilized and leftover has a negative ecological effect by leaching (Galloway et al., 2008). Controlled-release urea (CU) is designed to disperse N at rates that are synchronous with the N requirements of plants and is used as a beneficial, mitigating fertilizer alternative to reduce environmental pollution (Liu et al., 2019). Some studies have shown that CU decreases residual ${\mathrm{NO}}_{3}^{-}$ -N in the deep soil layer, decreases N losses resulting from ammonia volatilization, and increases the amount of N taken up by plants (Tang et al., 2021). Additionally, a one-time application of CU was found to save more labor and time than a conventional urea application. Although many CU-associated advantages have been confirmed by a considerable amount of research, large-scale CU applications to cereal crops have been limited, especially in developing countries. Most coating materials are derived from petroleum resources, which are expensive and nonrenewable (Doran, 2002). Moreover, coating shells may pose potential pollution risks to the soil environment.

To lessen the negative environmental and economic impacts of chemical fertilizer use, combining chemical fertilizers with organic fertilizers (OFs) is an essential strategy for sustainable agriculture (Muhammad et al., 2020). The raw materials used for OF preparation are abundant, and the cost of these materials is low. The application of OF can alleviate soil degradation and rebuild healthy soil. A previous study found that OF substitutions reduced the soil bulk density compared with that of non-OF fertilized soil (Blanco-Canqui et al., 2015), promoted soil fertility by increasing the contents of organic matter and other mineral nutrients (Meade et al., 2011), and accelerated the activities of beneficial microorganisms (Yadav et al., 2000). The decomposition of OF releases mainly N, which can increase wheat yields where the soil N supply is limited (Palmer et al., 2017). However, organic carbon sequestration potential can be changed by many factors, such as soil conditions and climate. If organic fertilizer is applied alone, the nutrients are released slowly, and the release period is long (Garzon et al., 2011), leading to the slow and variable short-term effects of OF applications on wheat yields. Thus, most farmers adhere to the use of synthetic fertilizers rather than OF to preserve their crop yields.

Partially substituting N fertilizer with OF makes it possible to take advantage of both the total nutrients and available nutrients, thus promoting biological activities in soils and the physicochemical characteristics of soils. Considering the cost and security of CU and the residual effect of OF, the combined application of OF with CU is hypothesized to improve wheat yields by releasing N synchronously with the N requirements of plants. The major objectives of the 2-year field experiment conducted in this study were to evaluate the effects of different fertilization treatments on wheat yield and NUE, to assess the environmental pollution risk induced by the leaching of inorganic N, and to identify the optimum ratio and feasibility of the applied OF and CU combinations. The experimental results provide a theoretical basis for scientific fertilization practices that consider both environmental and agronomic benefits in winter wheat fields.

## Materials and Methods

### Site and Material Descriptions

**Figure 1:**
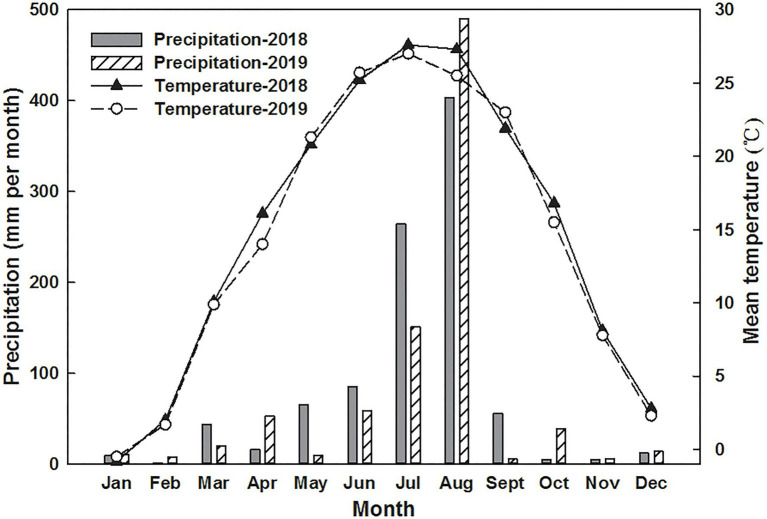
Variations in precipitation and air temperature.

A 2-year field experiment was established from October 2017 to June 2019 in the Yimeng Mountain area, Linyi, Shandong Province, China (35°7′4″N, 118°16′50″E). This area is a typical representative northern rocky mountain area with gravel, shallow soil, and low soil water storage capacity, and the soil depth is generally below 80 cm. The total precipitation amounts in 2018 and 2019 were 966.3 mm and 864.6 mm, respectively, and the average temperatures were 14.83°C and 14.43°C during the first and second growing seasons, respectively (Figure 1). The study area has cold winters and hot summers, and the rainy season generally spans from June to August (Yang et al., 2021). Before this study, the field was previously managed as a winter wheat/summer maize rotation system and had been continuously cultivated for more than 6 years. The annual N-P_2_O_5_-K_2_O fertilization rate was 400–300–350 kg ha^−1^, and no straw returning measures were adopted in this region. By request, the farmer did not implement summer fertilization of maize (N-P_2_O_5_-K_2_O was 210–150–180 kg ha^−1^) to reduce soil mineral N levels before beginning experiments.

The soil texture is classified as silty clay-loam, and the clay, sand and silt contents are 17.6, 15.8, and 66.6%, respectively. The chemical properties of the cultivated soil layer (0–20 cm) were as follows: total N, 0.88 g kg^−1^; organic matter, 12.5 g kg^−1^; total carbon, 10.1 g kg^−1^; ${\mathrm{NO}}_{3}^{-}$ -N, 68.4 mg kg^−1^; ${\mathrm{NH}}_{4}^{+}$ -N, 39.5 mg kg^−1^; available phosphorus, 35.0 mg kg^−1^; available potassium, 104.5 mg kg^−1^; and pH, 6.3.

The chemical fertilizers applied in the study area included concentrated superphosphate (P_2_O_5_, 46%), potassium chloride (K_2_O, 60%), urea (N, 46%), CU, sulfur-coated urea with a polymer coating, and 35% N, with a longevity of approximately 3 months. The morphology of the material before and after burial in soil was characterized by SEM and is shown in Supplementary Figure S1. The raw organic fertilizer material was cassava (cassava was used to produce ethanol, and the remaining waste residue was sufficiently decomposed to form organic fertilizer), which contained 60% organic matter with average nutrient contents of 2.5% N, 1.2% P_2_O_5_, 2.1% K_2_O and a pH of 6.8. FTIR spectra of organic fertilizer are shown in Supplementary Figure S2. All fertilizers were provided by Jin Yimeng Group CO., LTD, Shandong, China.

### Experimental Design and Field Management

The treatments were arranged in a randomized block design and repeated in triplicate. Six treatments were created: no N fertilizer (CK); urea providing 100% of the chemical N (urea); CU providing 100% of the chemical N (CU); organic fertilizer providing 30% of the N and CU providing 70% of the N (30%OF+70%CU); organic fertilizer providing 50% of the N and CU providing 50% of the N (50%OF+50%CU); and organic fertilizer providing 70% of the N and CU providing 30% of the N (70%OF+30%CU). All the fertilization treatments contained the same amount of N-P_2_O_5_-K_2_O (180–150–150 kg ha^−1^) according to the conventional recommended fertilizer rate in the local region. Before seeds were planted, organic and chemical fertilizers (except urea) were deposited as basal applications, and all fertilizers were incorporated into the soil at a depth of 10–15 cm. Urea (40%) was applied before planting, and urea (60%) was applied during the jointing period. The length and width of each experimental plot were 6 m and 5 m, respectively, and longitudinal and lateral protection areas of 0.5 m were placed around the plots to reduce marginal effects.

The cropping system was a winter wheat–summer maize rotation system, and the wheat and maize cultivars were “Jimai 22” and “Zhengdan 958,” respectively. Summer maize underwent similar treatments as wheat; in contrast, the amount of N-P_2_O_5_-K_2_O was 210–150–180 kg ha^−1^. Wheat was sown on October 4, 2017, and October 5, 2018, and harvested on June 5, 2018 and May 26, 2019, immediately followed by the sowing of maize in mid-June. Before planting winter wheat, the field was ploughed to a depth of 15–20 cm and rotary-cultivated to prepare the wheat seed bed. Seeds (150 and 165 kg ha^−1^) were planted in each plot in 2017 and 2018 at sowing depths of approximately 2–3 cm at a row spacing of 15 cm and subsequently flood irrigated. Depending on rainfall events, the field was flood-irrigated again in spring. As the soil moisture content was high in 2019, more wheat seeds were sown to ensure that the seedlings emerged evenly.

### Sampling and Analysis

#### Determination of the Yield, N Uptake, NUE and Economic Benefit Analysis of Wheat

Ten representative wheat plants were collected from each plot by cutting the aboveground portion at the seedling stage, jointing stage, grain-filling stage, and mature stage in 2019. At the mature stage of each year, the grain yield of wheat was determined using a 4 m^2^ (2 × 2 m) area in the center of each plot (adjusted to a 13% water content), and the thousand-grain weight was randomly calculated from the harvested grain. The straw biomass (stems and leaves) and grain were separated, and each component was dried at 105°C for half an hour and then dried to a constant weight at 75°C. Then, the samples were ground and passed through a 1-mm mesh screen for the following analyses. The total N contents of the straw and grain samples were obtained by digestion with H_2_SO_4_-H_2_O_2_-miscible liquids and determined by a Kjeldahl nitrogen analyzer (Douglas et al., 1980). The plant N uptake was calculated based on the dry matter weight and N concentration of each plant part.

The fertilizer NUE included the apparent recovery N use efficiency (ARNUE, %), agronomic nitrogen use efficiency (ANUE, kg kg^−1^) and partial factor productivity (PFP, kg kg^−1^). These data were calculated as described by Yang et al. (2021) using the following formulas:


$$ARNUE\left(\%\right)=\frac{N-N0}{F}\times 100$$ ( 1 )

$$ARNUE=\frac{Y-Y0}{F}$$ ( 2 )

$$PFP=\frac{Y}{F}$$ ( 3 )

In the above equations, *N* and *Y* represent the N uptake and grain yield obtained from N-treated plants, respectively, *N*0 and *Y*0 represent the N uptake and grain yield obtained from non-N-treated plants, respectively, and *F* represents the application rate of N.

The price of fertilizers was as follows: OF—62.5 US $ ha^−1^, CU—515.3 $ ha^−1^, potassium chloride—470 $ ha^−1^, urea—360 $ ha^−1^, potassium chloride—470 $ ha^−1^, and concentrated superphosphate—563 $ ha^−1^. The wheat grain yield was 406.9 $ ha^-1,^ and the labor cost of fertilization one time was 72.5 $ ha^−1^. The total revenue ($ ha^−1^) was the product of wheat grain yield and its price, and the net profit was calculated as the total revenue minus the total cost.

#### Release Characterization of CU

The N release characteristics of CU were detected by the method described by Geng et al. (2015). Briefly, 25 mesh bags (10 × 8 cm) containing 10 g CU granules were buried in the soil before sowing, and 3 bags were randomly selected each month, rinsed with distilled water to remove the loosely adhered soil, and dried. The N release rate from CU was calculated by measuring the weight loss.

### Soil Sampling and Analysis

Five soil samples were collected randomly from each plot and thoroughly mixed to produce a composite sample. Soil samples were collected with a soil corer at the seedling stage (on November 5, 2017, and November 11, 2018), jointing stage (on March 18, 2018, and March 16, 2019), grain-filling stage (on May 4, 2018, and May 2, 2019) and mature stage. The cultivated horizon of the 0–20-cm soil layer was sampled in both years, and soil samples were collected at depths of 0–100 cm at an increment of 20 cm at the seedling and mature stages in 2019. Then, the samples were air-dried and passed through 2-mm and 0.25-mm mesh sieves. The soil ${\mathrm{NO}}_{3}^{-}$ -N and ${\mathrm{NH}}_{4}^{+}$ -N were extracted with 0.01 mol L^−1^ KCl (the ratio of solution to soil was 10:1) and analyzed through a flow injection autoanalyzer (Bran-Luebbe, Norderstedt, Germany). The total N and total soil C concentrations were determined through combustion with an automatic elemental analyzer (Vario Micro Cube elemental analyser, Germany).

### Statistical Analyses

The raw data were preconditioned using Microsoft Excel 2017, and then the data were submitted to normalization tests and variance homogeneity tests. One-way ANOVA was used to assess the significant differences in total N, total C, ${\mathrm{NH}}_{4}^{+}$ -N, and ${\mathrm{NO}}_{3}^{-}$ -N. Two-way ANOVA was adopted to determine the effect of the interaction of year and N treatments on yield, yield component, and NUE. ANOVA and mean separation tests (Duncan’s multiple range test, at the 5% probability level) were performed using Statistical Analysis System package version 9.2 (2010, SAS Institute Cary, NC). All of the illustrations were drawn in SigmaPlot version 12.0 (MMIV Systat Software, Inc., San Jose, CA).

## Results

### Biomass and Grain Yield of Wheat

The statistical analysis showed that the total biomass strongly increased following N fertilization (Table 1), but the N sources had no obvious effect on the straw biomass. The total biomass, straw biomass, and grain yield showed significant differences between the 2 years, and these indexes were also affected by the interaction of N sources and year. The total biomass measured following the urea treatment was lower than those following the other N fertilization treatments in 2018, and the highest total biomass occurred in association with the 50%OF+50%CU treatment in 2019, but no obvious differences were observed among the other treatments. The effect of N application on the grain yield was greater than that on the straw biomass. Specifically, the straw biomass measured following the CK and urea treatments showed no remarkable differences in the first year, and no significant differences were detected among any N fertilization treatments in the second year. The grain yield decreased progressively with the OF dose in 2018, and the highest yield was observed in association with the 50%OF+50%CU treatment in 2019, reaching 8984.7 kg ha^−1^, 1.6–9.2% higher than that associated with the urea treatment; no significant differences were observed between the CU and OF+CU treatments. The thousand-grain weights varied from 38.5–42.4 g, and no pronounced difference was observed among treatments. (However, in 2019, the CK treatment resulted in a higher thousand-grain rate than the urea treatment.)

**Table 1:** Grain yields and straw biomass of wheat under different N application treatments.

Year	Treatment	Total biomass kg ha^−1^	Straw biomass kg ha^−1^	Grain yield kg ha^−1^	Thousand grain weight/g	Increase rate of grain vs. urea/%
2018	CK	14878.3c	7818.3c	7060.0c	40.6a	−8.41
	Urea	16144.8b	8436.3bc	7708.5b	40.4a	0
	CU	18376.9a	1036.0ab	8340.8a	39.7a	8.2
	30%OF+70%CU	18980.8a	10565.4a	8415.4a	40.0a	9.17
	50%OF+50%CU	18606.7a	10313.4a	8293.3ab	39.5a	7.59
	70%OF+30%CU	18782.8a	10954.4a	7828.4b	38.5a	1.56
2019	CK	15647.8c	8168.3b	7479.4c	43.4a	−9.75
	Urea	20190.1b	11902.6a	8287.5b	40.62b	0
	CU	21346.5ab	12850.4a	8496.0ab	41.7ab	2.52
	30%OF+70%CU	21247.3ab	12615.1a	8632.2a	40.9b	4.16
	50%OF+50%CU	22835.2a	13850.6a	8984.7ab	40.8b	8.41
	70%OF+30%CU	22291.4ab	13831.9a	8459.5ab	41.4ab	2.08
***Source of variation***						
N source		0.2817^*^	0.7959^ns^	0.2429^*^	0.1328^*^	
Year		0.3293^*^	0.1672^*^	0.2367^*^	0.6251^ns^	
N source × Year		0.1411^*^	0.01^*^	0.1631^*^	0.6978^ns^	

*The means followed by the same letter within a row are not significantly different at p < 0.05. “ns” means no significant difference*.

* *significant at 5%*.

### Nitrogen Uptake and N Use Efficiency

**Figure 2:**
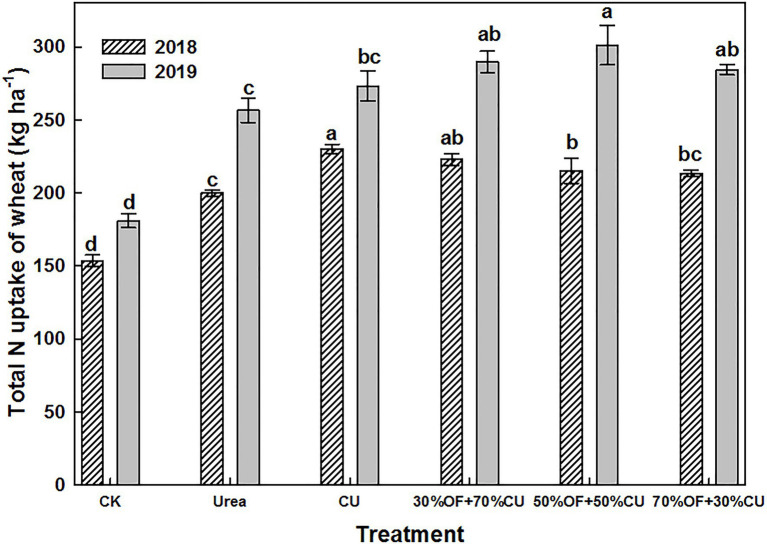
The total N uptake of wheat plants. The error bars indicate the standard errors (*n* = 3). The different lowercase letters above error bars indicate significant differences at the *p* < 0.05 level for each treatment in the same year.

The total biomass was higher in 2019 than in 2018, as was the total N uptake by the wheat plants (Figure 2). The combined OF and CU treatments showed some superiority in increasing N uptake compared with the urea and CU treatments, and the highest plant N uptake value appeared in the 50%OF+50%CU treatment in 2019. The ARNUE and ANUE were significantly affected by the N source, year, and the interaction of N source × year, while the PFP was not influenced by the interaction of N source × year (Table 2). The PFP values were not affected by the N fertilizer types, maintaining a wide range from 42.8–49.9 kg grain per kg N. However, the ARNUE was increased by combining OF and CU and was 29.2–58.9% higher in the OF+CU treatments than in the urea treatment. The ARNUE associated with the CU treatment was higher than those measured under the 50%OF+50%CU and 70%OF+30%CU treatments in 2018, but no significant differences were exhibited between the CU treatment and the OF+CU treatments in 2019. The maximum ARNUE values were 42.5% (CU) in 2018 and 66.7% (50%OF+50%CU) in 2019. No significant differences in ANUE were identified among the OF+CU treatments, but the values measured under the 50%OF+50%CU treatment were all higher than those associated with the urea treatment in both years of study.

**Table 2:** Nitrogen use efficiencies of wheat plants measured under different N application treatments.

Year	Treatment	ARNUE/%	PFP kg kg^−1^	ANUE kg kg^−1^
2018	Urea	25.73c	42.83a	3.60b
	CRU	42.47a	46.34a	7.67a
	30%OF+70%CRU	38.49ab	46.38a	8.09a
	50%OF+50%CRU	34.15b	46.07a	6.85a
	70%OF+30%CRU	33.22b	43.49a	4.27b
2019	Urea	41.97b	46.04a	4.49b
	CRU	51.28ab	47.2a	5.65ab
	30%OF+70%CRU	60.36a	47.96a	6.41ab
	50%OF+50%CRU	66.69a	49.92a	8.03a
	70%OF+30%CRU	57.41ab	47.0a	5.45ab
***Source of variation***				
N source		0.2819^*^	0.3509^*^	0.1741^*^
Year		0.5495^*^	0.2384^*^	0.0835^*^
N source × Year		0.1594^*^	0.8890^ns^	0.3227^*^

*ARNUE, apparent recovery nitrogen use efficiency; PFP, partial factor productivity; ANUE, agronomic nitrogen use efficiency*.

* *significant at 5%*.

### Economic Benefit Analysis of Wheat

Urea was used as basal and topdressing fertilizer, leading to double the labor cost of fertilization than the other fertilizers (Table 3). The total revenue and net profit of CK were the lowest in both years. No obvious difference of total revenue was exhibited among the CU, 30%OF+70%CU and 50%OF+50%CU treatments, but it was significant higher in the three treatments than that in the 70%OF+30%CU and urea treatments in 2018; meanwhile, the total revenues of the 50%OF+50%CU and 30%OF+70%CU treatments were higher than that of the urea treatment in 2019. Similarly, the net profit of the 50%OF+50%CU treatment was prominently higher than that of the urea treatment by 11.9 and 12.7%, respectively, in 2018 and 2019, but there was no significant difference between the CU and OF treatments.

**Table 3:** Average cost, total revenue, and net profit of wheat under different N application treatments ($ ha^−1^).

Treatment	Fertilizer cost	Labor cost of fertilization	Other costs	Total revenue		Net profit	
				2018	2019	2018	2019
CK	301.4	72.5	700	2872.7c	3043.4c	1798.8b	1969.5c
Urea	397.4	145	700	3136.6b	3372.2b	1894.2ab	2129.8b
CU	474.9	72.5	700	3393.9a	3457.0ab	2146.5a	2209.6ab
30%OF+70%CU	479.7	72.5	700	3424.2a	3512.4a	2172.0a	2260.3ab
50%OF+50%CU	483.0	72.5	700	3374.5a	3655.9a	2119.0a	2400.4a
70%OF+30%CU	486.3	72.5	700	3185.4b	3442.2ab	1926.6ab	2183.4ab

*Other costs include seeds, machinery, pesticides, insecticides, irrigation, and other materials and expenses*.

### Biomass and Total N Uptake Dynamics of Wheat Plants

**Figure 3:**
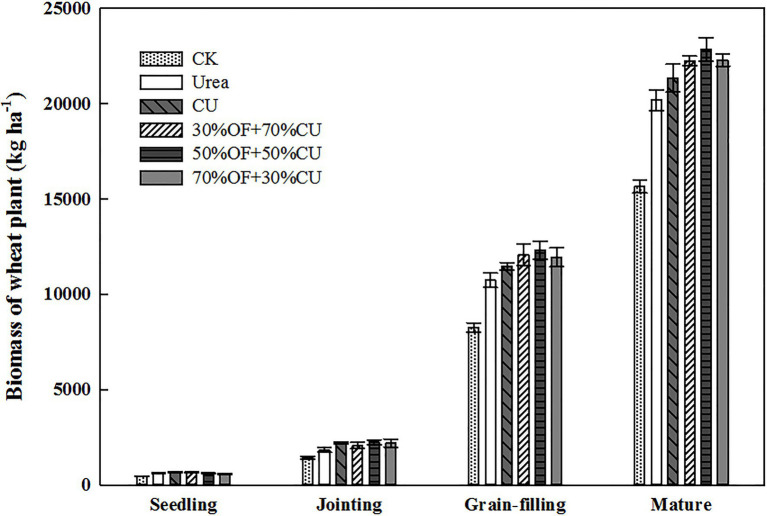
Biomass dynamics of wheat plants during the growing season.

**Figure 4:**
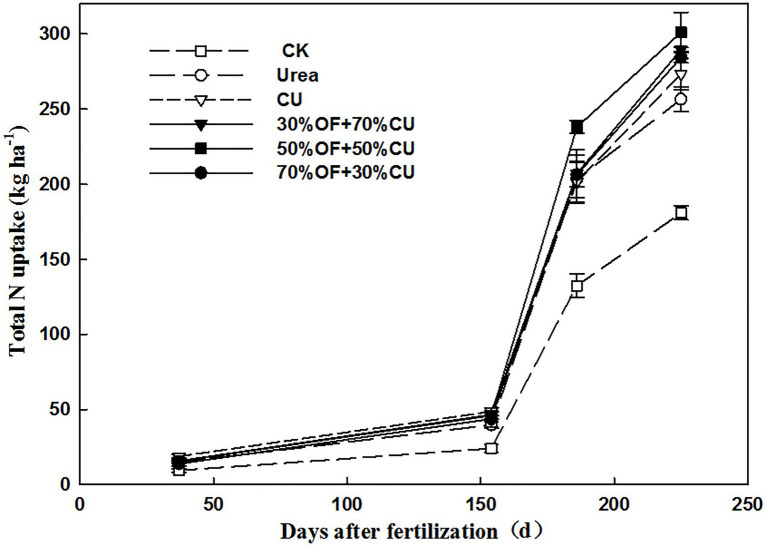
Total N uptake of wheat plants during the growing season.

The biomass (Figure 3) and total N uptake (Figure 4) increased with increasing growth period, and the two indicators under the CK treatment exhibited the lowest values in each stage. Specifically, the biomass ranged from 579.5 to 681.0 kg ha^−1^, and no significant difference was found among all N-applied treatments (except CU, which was higher than that in 50%OF+50%CU) in the seedling stage. Although there was 117 d from the seedling stage to the jointing stage, the wheat biomass did not increase much, as it was winter, and the weather was cold. In the grain-filling stage, biomass was significantly increased by OF application, and the values in the OF treatments were higher than those in the CU and urea treatments. Moreover, the values in the CU treatment were larger than those in the urea treatment. At the mature stage, the highest and lowest plant biomass appeared in the 50%OF+50%CU and urea treatments, which reached 22835.2 and 20190.1 kg ha^−1^, respectively. Total N uptake dynamics showed similar trends with biomass.

### Release Characterization of CU

**Figure 5:**
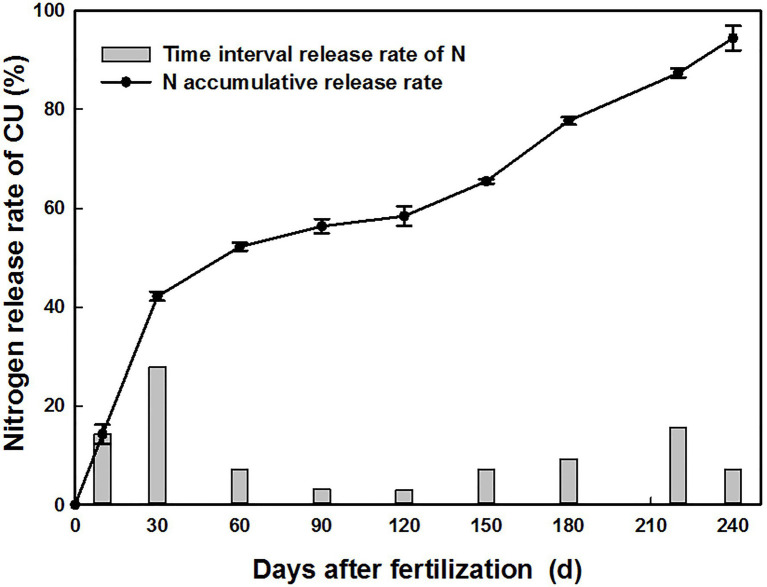
Release characteristics of CU in soil.

The N within CU was released quickly in the first 2 months of the study period, with N releases of 14.3, 42.2, and 52.2% measured on the 10th, 30th and 60th days after burial in soil, respectively; only 13.2% of the total N was released from the 60th to the 150th day. Then, the release rate gradually increased from the 150th to the 220th day, and 94.4% of N was released at the time of harvest (Figure 5).

### Temporal Variations in the Soil Total Carbon Content and Soil Bulk Density

**Figure 6:**
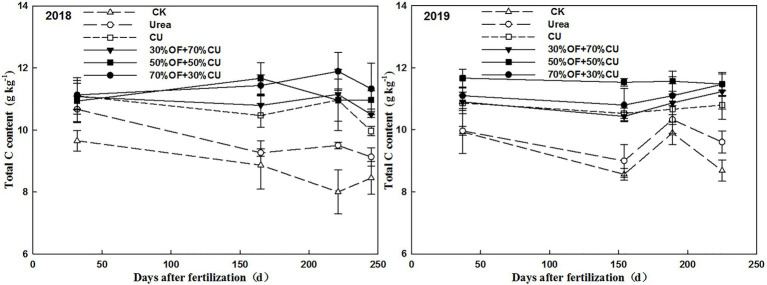
Variations in the total carbon concentrations of the 0–20-cm topsoil.

The lowest total carbon level was observed in the CK treatment at each ontogenetic stage in both years (Figure 6). The total carbon content increased with an increasing OF application rate in the first year, but the 50%OF+50%CU treatment yielded the highest value in 2019. The total carbon content associated with the urea treatment was lower than those of the other N application treatments, and CU showed no significant difference from 30%OF+70%CU from the seedling stage to the grain-filling stage. Nevertheless, the total carbon content was lower in the CU treatment than in the OF+CU treatments at the mature stage. No obvious difference in soil bulk density was exhibited among all treatments (Supplementary Figure S3).

### Temporal Variation in the Soil Total Nitrogen Content

**Figure 7:**
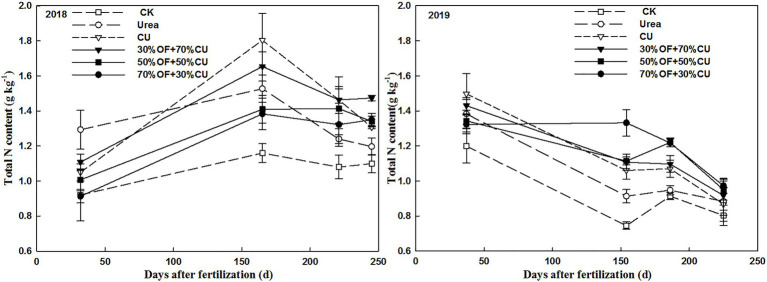
Variations in the total N concentrations of the 0–20-cm topsoil.

Generally, in 2018, the total N content showed a tendency of first increasing from the seedling to the jointing sage and then decreasing; in 2019, it displayed a roughly downward trend (Figure 7). Specifically, in 2018, the total N content measured in the urea treatment was higher than those measured in other treatments in the seedling stage but was lower beginning in the jointing stage. The values decreased with the increasing OF substitution rate in the OF+CU treatments, and 70%OF+30%CU showed no significant difference from CK in the seedling stage. At harvest, the highest value was observed under the 30%OF+70%CU treatment, and no significant difference was found among 50%OF+50%CU, 30%OF+70%CU, or CU. In 2019, the lowest values were still exhibited in association with the CK treatment at each stage of growth, and the greatest levels appeared under the CU, 70%OF+30%CU and 50%OF+50%CU treatments at the seedling, jointing, and grain-filling stages, respectively. In addition, no significant difference was observed among the OF + CU treatments at harvest.

### Temporal Variations in the Soil NO3– -N and NH4+ -N Contents

**Figure 8:**
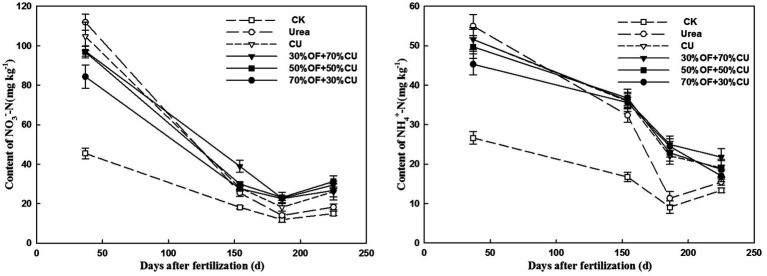
Variations in ${\mathrm{NO}}_{3}^{-}$ -N and ${\mathrm{NH}}_{4}^{+}$ -N in the 0–20-cm topsoil in 2018.

In all treatments, the temporal variations in the ${\mathrm{NO}}_{3}^{-}$ -N and ${\mathrm{NH}}_{4}^{+}$ -N concentrations showed downward trends throughout the growth stages (Figure 8), and these concentrations were markedly improved by N fertilization compared with the CK treatment. The levels of inorganic N differed significantly among the N-applied treatments. The highest ${\mathrm{NO}}_{3}^{-}$ -N and ${\mathrm{NH}}_{4}^{+}$ -N values were yielded under the urea treatment at the seedling stage, reaching 111.9 mg kg^−1^ and 55.0 mg kg^−1^, respectively. However, these values dropped dramatically from the jointing stage and were significantly lower than the values measured under other N-applied treatments. Under the CU treatment, the ${\mathrm{NO}}_{3}^{-}$ -N and ${\mathrm{NH}}_{4}^{+}$ -N values were lower than those measured under the OF+CU treatments, except in the seedling stage. The ${\mathrm{NO}}_{3}^{-}$ -N and ${\mathrm{NH}}_{4}^{+}$ -N concentrations were significantly increased by OF application, especially from the grain-filling to mature stages.

### Leaching of Nitrate Nitrogen and Ammonium Nitrogen With Time in the 0–100-cm Soil Layer Two Years After Fertilization

**Figure 9:**
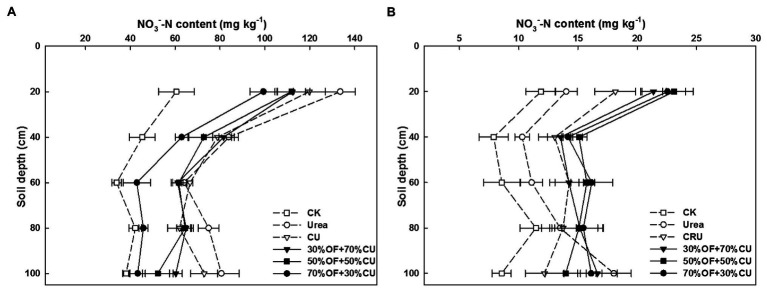
The distributions of soil ${\mathrm{NO}}_{3}^{-}$ -N in the 0–100-cm soil profiles at the seedling stage **(A)** and mature stage **(B)** in 2019.

The lowest ${\mathrm{NO}}_{3}^{-}$ -N level was observed under the CK treatment in each soil layer, regardless of the growth stage (Figures 9A,B). In addition, the ${\mathrm{NO}}_{3}^{-}$ -N level exhibited a general downward trend as the soil depth increased under the CK treatment, except for the peak observed in the 60–80-cm layer. The ${\mathrm{NO}}_{3}^{-}$ -N concentrations measured under all treatments were higher at the seedling stage than at maturity. In contrast, these values were lower in the OF+CU treatments than in the urea treatment 37 days after fertilization in the 0–40-cm soil layer, but the opposite results appeared at maturity. In the seedling stage, the greatest values in the 60–100-cm soil layers were detected under the urea treatment, and the value measured under the urea treatment was also higher in the 80–100-cm soil layer at maturity. Moreover, at maturity, the amounts of nitrate N in the 0–100-cm soils were higher in the OF+CU treatments than in treatments that used CU alone. The 50%OF+50%CU treatment presented a higher level in the 0–60-cm soil layer than those of 30%OF+70%CU and 70%OF+30%CU.

**Figure 10:**
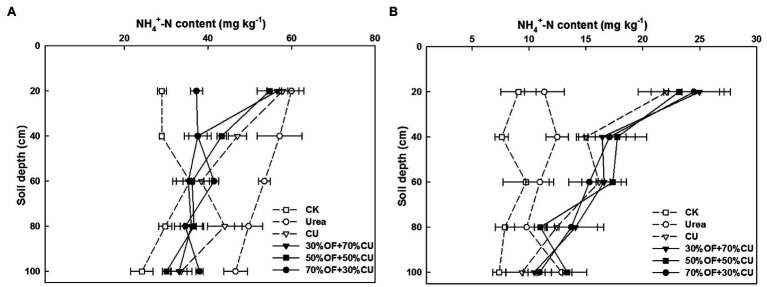
The distributions of soil ${\mathrm{NH}}_{4}^{+}$ -N in the 0–100-cm soil profiles at the seedling stage **(A)** and mature stage **(B)** in 2019.

With wheat growth, the ${\mathrm{NH}}_{4}^{+}$ -N content decreased from the seedling stage (Figure 10A) to maturity (Figure 10B). Under each treatment, the variation trend of ammonium N was roughly consistent with that of nitrate N. By using urea, the soil ${\mathrm{NH}}_{4}^{+}$ -N content in the seedling stage increased at depths of 0–100 cm but was lower at the 0–80-cm depths at maturity. No pronounced difference was found among OF+CU treatments at 0–60-cm depths; nevertheless, all these treatments yielded higher values at 0–40-cm depths at maturity than those measured under the CU treatment.

## Discussion

### Combined Effects of OF and CU on Wheat Yield and Nitrogen Use Efficiency

Excessive chemical N fertilization and improper fertilizer application practices intensify N losses and result in low N utilization rates (Pinochet et al., 2018), which can be attributed to the asynchrony between wheat N deficits and N fertilizer applications (Osterholz et al., 2017). In the current experiment, the contribution of the grain weight to the total biomass was remarkable compared with the contribution of the straw weight. The ARNUE was higher in 2019 than in 2018 due to the higher initial fertility of the tested soil; otherwise, the differences in precipitation between the two years also contributed to the difference in results; this result was consistent with the observations reported in previous research (Geng et al., 2016). Compared with CU, the rapid hydrolysis of urea resulted in a higher concentration of inorganic N in the early stage, but this treatment could not produce a sustained N supply until the grain-filling stage (Varinderpal-Singh et al., 2020).

At the early growth stages, the poor absorption and interception abilities of wheat roots restricted N assimilation and may have accelerated the amount of reactive N escaping or leaching from fertilizer N (Oka et al., 2012). Optimal wheat yields depend on the agronomic capacity to synchronize the N availability in soils with the N demands of plants (Clunes and Pinochet, 2020). Wheat can even be successfully grown without N fertilizer use at sowing in soils with indigenous N supplies (Liu et al., 2017). The N within CU was released quickly in the first 2 months of the study and reached 42.2% in the first month, suggesting that the N supply from CU was sufficient for wheat growth at the early ontogenetic stage. The continuous release of N in the later stages also played an important role in increasing the yield, thus increasing the NUE. Moreover, the sulfur polymer-coated films partially degraded, signifying that the CU coating materials did not cause secondary pollution to the soil environment.

The functional groups of OF did not change extensively before or after the composting process, and the absorption peaks observed at 1636 and 3,416 cm^−1^ can be attributed to the stretching vibrations of the C=C and O-H bonds in the aromatic rings (Kumar et al., 2013). The presence of the C-O(H) group resulted in the observed peak at 1034 cm^−1^, and CH_2_ and C-H scissoring vibrations were observed at 2923 and 1,401 cm^−1^. Intricate functional groups made the residual effects of OF application visible, and the continuous supply of N led to higher nutrient availabilities for wheat growth. Most of the N in OF existed in organic forms, resulting in a slow release of available N, and failed to meet the N requirement of wheat plants, thus leading to lower biomass in the early growth stage. Then, the organic matter from OF gradually decomposed and mineralized more nutrients, such as N, P and K, since the extension of the fertilization period and increased rainfall (Jiang et al., 2020). It continued supplying nutrients to the wheat during the later growth stage or next year of production. Therefore, the increased production is not only caused by N but also depends on the content of organic matter in the soil.

Higher organic carbon contents were yielded by applying OF than by applying conventional urea; these results were analogous with the findings of Sihi et al. (2017). When the amount of organic carbon in soil increases, wheat grain yield production increases concurrently. Integrated fertilization methods using both CU and OF were shown to be effective for improving wheat productivity. Compared with urea, the 70% OF substitution did not further promote the wheat yield compared with the wheat yield measured under the urea-only fertilization treatment. A high OF application rate results in an apparent increasing effect on the wheat yield but a weaker increasing effect than that induced by urea application and an even lower effect than that observed through CU application. These results may be attributed to the residual effect of OF application; the residual effect was found to maintain crop yields for several years in long-term experiments (Shen et al., 2007), while a high OF ratio was not an ideal model for increasing crop yields in short-term management studies. Under field conditions, crop growth depends mainly on available mineral nutrients, and the yield-increasing effect of organic fertilizer was not significant in the studied growth season.

Although the use of OF has contributed prominently to environmental sustainability and increased crop production (Osterholz et al., 2017), OF has not been used extensively in China. The incorporation of OF with CU presents a viable alternative to standard chemical N fertilizer, as the slow and variable short-term effects of OF could be averted and could be made full use of the advantages of CU. Thus, the uptake of N induced by substituting OF could be increased, supporting the achievement of higher biomass and grain yields. Although OF combined with CU spend more input on fertilizer, the 50%OF+50%CU treatment achieved higher net profit by 11.9–12.7% compared with the urea treatment due to its higher grain yield. More importantly, top dressing is avoided, and manual labor is reduced by the combination of OF and CU, as the worker population is aging and labor shortages for crop-production activities are becoming increasingly severe. The interannual variation in precipitation is large both temporally and spatially, leading to different rates of decomposition and mineralization of OF, thus affecting wheat yield and the distribution of ${\mathrm{NO}}_{3}^{-}$ -N in soil. However, the combined application of OF with CU showed an increasing trend with different increase amplitudes in both years and promoted net profit. The interaction effect will be universal across regions and years, and this result is beneficial for updating the extension policy of OFs in China.

### Combined Effects of OF and CU on Nitrogen Leaching

Agriculture intensification and expansion demand high doses of chemical N fertilizer in cultivated lands (Fan et al., 2021), and the unprecedented input of N resulting from these fertilizer applications would increase N availability and cycling and, subsequently, ${\mathrm{NO}}_{3}^{-}$ -N leaching from agroecosystems (Galloway et al., 2008). The rapid hydrolysis of urea resulted in higher inorganic nitrogen contents being measured at the seedling stage, but root growth was slow at this time, and the wheat plants had small interception areas, thus limiting N uptake from fertilizer (Wu and Ma, 2015). Moreover, high soil ${\mathrm{NO}}_{3}^{-}$ -N and ${\mathrm{NH}}_{4}^{+}$ -N concentrations enhance soil fertility but also increase the risk of harmful nitrate leaching to the groundwater. The distributions of soil ${\mathrm{NO}}_{3}^{-}$ -N and ${\mathrm{NH}}_{4}^{+}$ -N in the 0–100-cm soil profiles confirmed this phenomenon.

Otherwise, the “luxury” N consumption that occurs when N is taken up by wheat is higher than the critical N concentration required before the reproductive growth stages (Liu et al., 2017), leading to straw biomass not differing prominently among N-treated management practices. For the same N input, the N in the substituted OF must be mineralized, resulting in the slowed release of mineral N (Gutser et al., 2005). Contemporarily, the N supplied by CU was released according to the N demands of wheat, and more mineral N was maintained in the topsoil, thus feeding plants and decreasing the leaching of N (Geng et al., 2016).

Large ${\mathrm{NO}}_{3}^{-}$ -N accumulation and leaching in the soil profile occurs as mineralized N well exceeds the N demands of crops (Yang et al., 2020). In the current study, ${\mathrm{NO}}_{3}^{-}$ -N and ${\mathrm{NH}}_{4}^{+}$ -N were mainly concentrated in the 0–60-cm soil layers in the OF substitution treatments, preventing N from leaching into deeper layers, and their appearance was enhanced as the rate of OF application increased. This may have been because OF can alter N cycling by providing nutrients to N cycling-related soil microbial communities (Thangarajan et al., 2013), improving the physicochemical properties of soil (Muhammad et al., 2020); this process may temporarily immobilize mineral N in the soil and contribute to reduced leaching losses. Otherwise, OF fertilization augmented soil organic carbon, and higher organic matter contents increased the abiotic sorption and biotic degradation processes of synthetic chemicals, resulting in lower chemical leaching (Levanon et al., 1993). In China, soil productivity has declined dramatically, and the environmental impact of reactive N has been evident for some time, as more synthetic N is applied (Hartmann et al., 2015). The economic net benefits in OF treatments were improved by 11.87–12.68% compared with urea, but OF had a residual effect and was beneficial for improving the quality of cultivated land, and the increment of wheat yield was more obvious over time. Meanwhile, for the same N input, the total N contents in topsoil of the OF substitution treatments were higher than those in the urea treatments, which indicated that more N was leached into the environment or deep soil of the urea treatment. Considering the environmental and agronomic benefits together, the combination of 30–50% OF with CU was the most conducive for enhancing wheat production.

## Conclusion

Combining OF and CU could regulate the continuous release of N from fertilizers to match the corresponding N uptake of wheat. The 30%OF+70%CU and 50%OF+50%CU treatments showed some superiority in increasing the grain yield and N uptake compared with the urea treatment, with increase rates of 4.2–9.2% and 6.7–17.3%, respectively. The net profit of the 50%OF+50%CU treatment was prominently higher than that of the urea treatment by 11.9–12.7%. ${\mathrm{NO}}_{3}^{-}$ -N and ${\mathrm{NH}}_{4}^{+}$ -N were mainly concentrated in the 0–60-cm soil layer and reduced N leaching to the 60–100-cm soil layer by the combined application of OF and CU. The application of OF is beneficial to the soil of the Yimeng Mountain area, not only in enhancing wheat productivity while mitigating the environmental pollution induced by N but also due to its residual effect. Undoubtedly, more research *via* long-term field experiments is still necessary.

## Data Availability Statement

The original contributions presented in the study are included in the article/Supplementary Material, further inquiries can be directed to the corresponding author.

## Author Contributions

XY: methodology, software, and writing. CZ: resources and investigation. XM: investigation. QL: conceptualization and formal analysis. JA: supervision. SX and XX: edit and revise. JG: project administration and validation. All authors contributed to the article and approved the submitted version.

## Funding

This research was supported by the Natural Science Foundation of China (Grant no. 42007091/42077061/41977067/41977262), Project of Introducing and Cultivating Young Talent in the Universities of Shandong Province (Soil Erosion Process and Ecological Regulation), and the Natural Science Foundation of Shandong Province of China (ZR2020QC163).

## Conflict of Interest

CZ and XM were employed by company Jinyimeng Group Co. Ltd.

The remaining authors declare that the research was conducted in the absence of any commercial or financial relationships that could be construed as a potential conflict of interest.

## Publisher’s Note

All claims expressed in this article are solely those of the authors and do not necessarily represent those of their affiliated organizations, or those of the publisher, the editors and the reviewers. Any product that may be evaluated in this article, or claim that may be made by its manufacturer, is not guaranteed or endorsed by the publisher.

## Supplementary Material

Supplementary Figure S1 SEM images of the surface morphology of CU films. Panels A1, A2, and A3 show the CU surfaces before fertilization at 5 μm, 10 μm, and 50 μm, respectively. Panels B1, B2 and B3 show the CU surfaces after burial in soil at 5 μm, 10 μm, and 50 μm, respectively.

Supplementary Figure S2 FTIR spectra of organic fertilizer.

Supplementary Figure S3 Bulk density of the 0–20-cm topsoil in 2019.
