# Supplementary figures and images for: Combining Organic Fertilizer With Controlled-Release Urea to Reduce Nitrogen Leaching and Promote Wheat Yields
Source: Front Plant Sci. 2021 Dec 24;12:802137. doi: 10.3389/fpls.2021.802137 (PMC8740327; doi:10.3389/fpls.2021.802137)

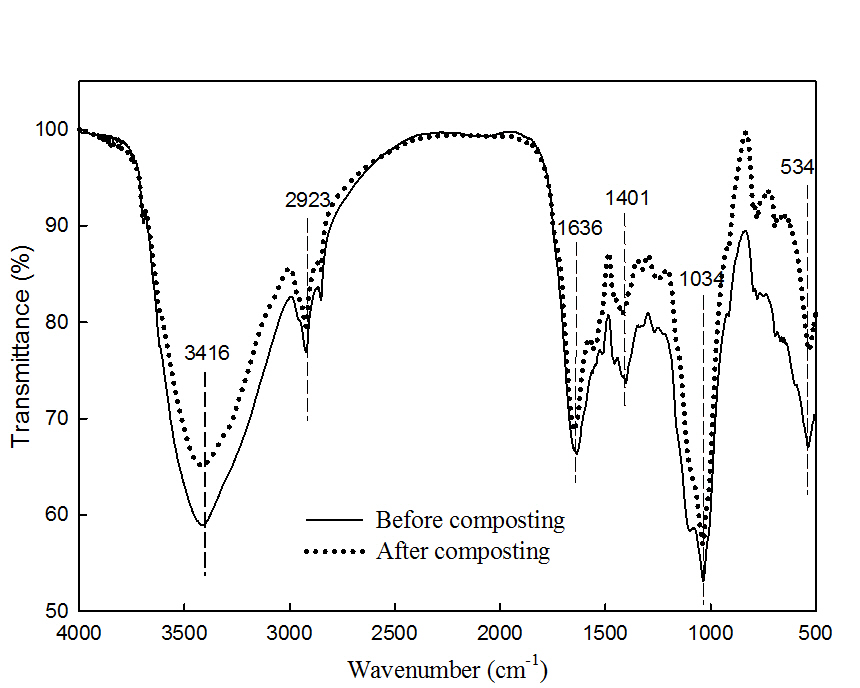

Supplement: Supplementary Figure S1 — SEM images of the surface morphology of CU films. Panels A1, A2, and A3 show the CU surfaces before fertilization at 5 μm, 10 μm, and 50 μm, respectively. Panels B1, B2 and B3 show the CU surfaces after burial in soil at 5 μm, 10 μm, and 50 μm, respectively. [file Image_1.JPEG]

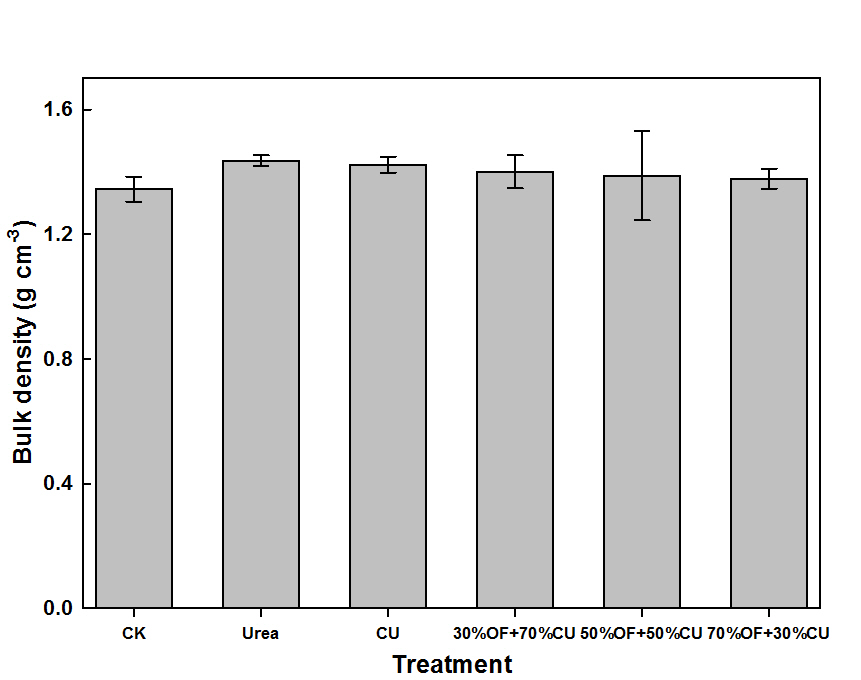

Supplement: Supplementary Figure S2 — FTIR spectra of organic fertilizer. [file Image_2.JPEG]

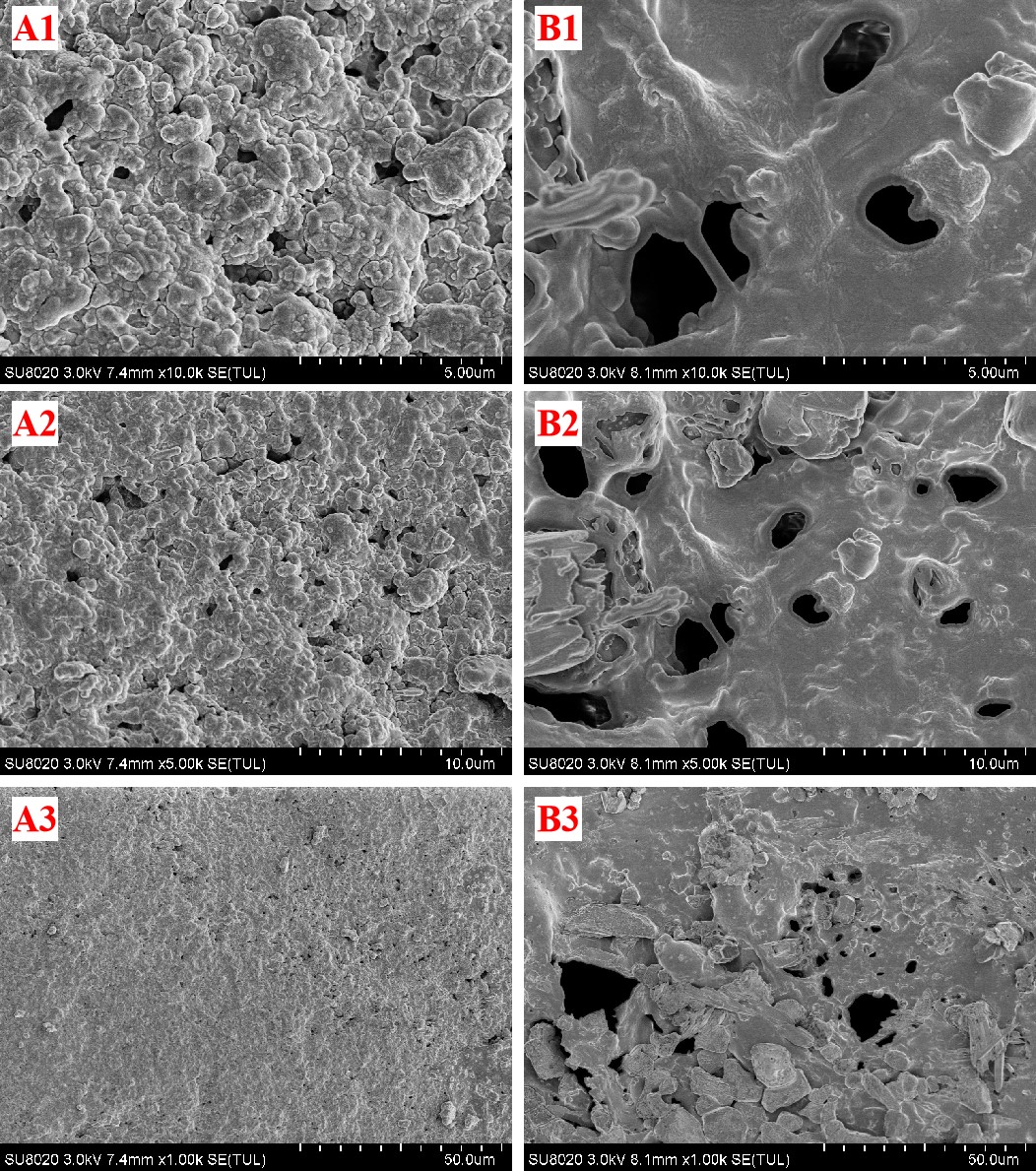

Supplement: Supplementary Figure S3 — Bulk density of the 0–20-cm topsoil in 2019. [file Image_3.JPEG]
